# Reassessing the risks and benefits of COVID-19 precautions in 2023

**DOI:** 10.18632/oncotarget.28468

**Published:** 2023-09-22

**Authors:** Thomas A. Ollila, Rashida Taher, Prashanth Moku

**Keywords:** COVID, immunosuppression, cancer

The COVID-19 pandemic has killed over one million Americans with many dying during the Omicron wave. By now most Americans have either had COVID-19 and/or been vaccinated against it. Despite the availability of updated immunizations, only 16.7% of Americans are now up-to-date on bivalent boosters [1]. At our cancer center, we treat many patients with hematologic malignancies, most of whom are older adults. Patients with hematologic malignancies, especially lymphoma, are at increased risk of poor response to vaccination and worse outcomes from COVID-19 infection [2]. Most of our patients have been abundantly cautious since the onset of the pandemic and some have avoided ever becoming infected. Patients in our clinic frequently inquire about the safety of being outdoors, spending time with their families during large gatherings (Christmas, Thanksgiving, etc.), and methods to prevent the contraction of COVID-19. Despite these precautions, too many patients reached remission from cancer only to then perish from COVID-19 in the first years of the pandemic. The concerns behind their questions are very real but understanding how to best answer them is not always easy, and their abundance of caution is not without cost. Grandchildren’s birthdays went uncelebrated, weddings were forgone, and memorable moments with loved ones were lost. With both aging and malignancy, an acute awareness of the limited days means that there may not be years ahead to make up for all that was missed. The balance between appropriate precautions and the harm of social isolation always requires a thorough appraisal.

“Alone Together” was a commonly used slogan during the initial phase of the COVID-19 lockdown in the spring of 2020. The world was facing an unprecedented period of social disengagement to slow the spread of the virus. The immediate need for social isolation was clear but the longer-term psychological sequelae were not. It has been postulated that suicide rates in the next several years will rise not only in the United States, but in other countries as well due to the development or exacerbation of depression, anxiety, and substance abuse related to social isolation [3]. Furthermore, research has consistently shown that the effects of social isolation extend beyond the psychological to the physical and contribute to higher rates of cardiovascular disease and type II diabetes [4]. Social isolation’s negative impact is even more profound among the elderly and those facing serious illnesses including cancer [5, 6]. Hence, the development and utilization of therapeutic agents directed against COVID-19 remains critical to allow for the liberalization of social restrictions and to ensure treatment options for those with severe infections.

In addition to increased immunity in the population through vaccines and infection, we have made tremendous advances in fighting serious illness and deaths from COVID-19. Antivirals remain effective against COVID-19 and provide a valuable treatment option. Two anti-viral agents are recommended as the preferred treatment of COVID-19: remdesivir and nirmatrelvir-ritonavir (Paxlovid) [7]. Remdesivir is administered intravenously and typically used in hospitalized patients, although it has been approved for both inpatient and outpatient settings. In separate trials, remdesivir has been shown to have significantly reduced 28-day hospitalization or death and also shown to have reduced time to recovery compared to placebo [7]. Paxlovid is an oral agent administered twice daily for five days. Patients frequently inquire about Paxlovid rebound, which is a known adverse effect. However, Paxlovid has been shown to reduce the risk of 28-day hospitalization or death by 89% compared to placebo in outpatients with COVID-19 who are at high risk of disease progression [7]. Hence, it is a very effective anti-viral option against COVID-19. When discussing its use and risk of rebound we acknowledge this while focusing on its benefit, which is to keep people out of the hospital and from dying from COVID-19. At this job, it excels. A third oral agent, molnupiravir is recommended as an alternative therapy if the aforementioned two agents are not available or clinically inadvisable [7, 8]. Notably, monoclonal antibodies (mAbs) have lost efficacy against COVID-19. In December 2022, the NIH COVID-19 Treatment Guidelines Panel recommended against the use of any mAbs due to the omicron variant that accounted for the majority of cases in the USA and was largely resistant to these agents [8]. In January 2023, the FDA revoked the emergency use authorization for the mAB Evusheld, the only agent authorized for pre-exposure prophylaxis, due to the overall prevalence of Omicron variants estimated to be >97% [8].

I look back at the challenging times of treating patients during the COVID-19 pandemic in a Hematology office. A typical day included patients’ family members crying over a speakerphone from the clinic parking garage as their loved ones formally received their cancer diagnoses. Medical students raced to set up iPads to allow patients to share their goodbye’s remotely with families. Patients who would otherwise strongly benefit from rehabilitation services after hospital discharge went home without adequate services; either due to concern over the outbreak, or a simple lack of available rehab centers Four years have passed since the emergence of COVID-19 and the world is still reeling from its effect. However, it is important to remember that COVID-19 is not the world’s first pandemic, and it is unlikely to be the last. In 2009, the globe was hit by the H1N1 influenza pandemic. In the century prior, pandemics occurred in 1918, 1957–1958, and 1968. The 1918 influenza pandemic is the most severe in recent history and is often compared to the current one. While there are similarities, there are also striking differences- the most important being the development of effective vaccines to protect individuals who become exposed to and/or infected with the virus. We also now have therapies that are authorized or approved to treat patients with mild-to-moderate symptoms who are considered at high risk for progression to severe illness and/or possibly death [7, 8]. With current national health and public health emergency declarations due to end in May 2023, it is imperative to continue individualized counseling for patients and their families. Although COVID-19 continues to pose a serious threat, medical advancements have now allowed for a more in-depth risk-benefit discussion to weigh the risk of infection versus the challenges of social isolation. Most of our counseling now focuses on liberalizing restrictions safely and vigilantly. We can now advise most of our clinic patients that it is okay to hug their grandchildren.
